# *HABP2* G534E Variant in Papillary Thyroid Carcinoma

**DOI:** 10.1371/journal.pone.0146315

**Published:** 2016-01-08

**Authors:** Jerneja Tomsic, Rebecca Fultz, Sandya Liyanarachchi, Huiling He, Leigha Senter, Albert de la Chapelle

**Affiliations:** 1 Department of Molecular Virology, Immunology and Medical Genetics, Ohio State University Wexner Medical Center and Comprehensive Cancer Center, Ohio State University, Columbus, Ohio, United States of America; 2 Department of Internal Medicine, Ohio State University Wexner Medical Center and Comprehensive Cancer Center, Ohio State University, Columbus, Ohio, United States of America; IPATIMUP/Faculty of Medicine of the University of Porto, PORTUGAL

## Abstract

The main nonmedullary form of thyroid cancer is papillary thyroid carcinoma (PTC) that accounts for 80–90% of all thyroid malignancies. Only 3–10% of PTC patients have a positive family history of PTC yet the familiality is one of the highest of all cancers as measured by case control studies. A handful of genes have been implicated accounting for a small fraction of this genetic predisposition. It was therefore of considerable interest that a mutation in the *HABP2* gene was recently implicated in familial PTC. The present work was undertaken to examine the extent of *HABP2* variant involvement in PTC. The *HABP2* G534E variant (rs7080536) was genotyped in blood DNA from 179 PTC families (one affected individual per family), 1160 sporadic PTC cases and 1395 controls. RNA expression of *HABP2* was tested by qPCR in RNA extracted from tumor and normal thyroid tissue from individuals that are homozygous wild-type or heterozygous for the variant. The variant was found to be present in 6.1% familial cases, 8.0% sporadic cases (2 individuals were homozygous for the variant) and 8.7% controls. The variant did not segregate with PTC in one large and 6 smaller families in which it occurred. In keeping with data from the literature and databases the expression of *HABP2* was highest in the liver, much lower in 3 other tested tissues (breast, kidney, brain) but not found in thyroid. Given these results showing lack of any involvement we suggest that the putative role of variant *HABP2* in PTC should be carefully scrutinized.

## Introduction

Thyroid cancer represents 3.8% of all new cancer cases in the United States. It is estimated that 62,450 new cases will be diagnosed in 2015 in the United States (www.seer.cancer.gov).

PTC has been shown to be one of the most heritable cancers of all [1–5]. It is therefore notable that only few gene variants have been suggested, let alone proven to predispose to PTC. In early reports linkage analyses pointed to several loci for genetic predisposition to PTC but causal variants have not been found [6]. More recently several studies based on the candidate gene approach have found variants associated with predisposition to PTC but the variants appear to be rare being found in just 1 family [7], present in a few families [8] or present in one family and a small number of “sporadic” cases [9,10]. Other variants emanate from genome-wide association studies (GWAS) but account for just some 10% of the estimated genetic predisposition [11]. In conclusion, the predisposition from all the above variants accounts for just a minority of all PTC.

Recently Gara et al. [12] reported a germline *HABP2* mutation causing familial nonmedullary thyroid cancer. This missense mutation (c.1601G>A; p.G534E; rs7080536) was found in a family affected with PTC and was shown to segregate with PTC in that family. The variant was present in 4.7% of 423 patients in the TCGA database and was reported to occur in 0.7% when combining controls from 1000 Genomes Project (phase 3; www.1000genomes.org) and HapMap3 (release 3; http://hapmap.ncbi.nlm.nih.gov/), suggesting a relatively high population attributable fraction. The present study was undertaken to elaborate further on the role of the *HABP2* variant in PTC risk.

## Materials and Methods

### Patients and controls

The studies were approved by the Institutional Review Board at The Ohio State University Medical Center. All subjects gave written informed consent before participation.

Individuals with thyroid cancer from The Ohio State University (OSU) collection were histologically confirmed as having traditional PTC or follicular variant PTC. The majority of the patients had no family history and were designated as “sporadic”. We defined “familial” broadly as the occurrence of two or more first- or second-degree relatives with PTC; there were in total 179 families meeting this criterion. Of these families 103 have 3 or more first or second degree relatives (Group 1) while 76 have only 2 individuals with PTC (Group 2). The majority of our families are of Caucasian origin. Information on sporadic cases and controls used in this study can be found in Tomsic et al. 2015 [10] and S1 Table.

### DNA and RNA extraction

Genomic DNA was extracted from blood following standard phenol-chloroform extraction procedures. RNA was extracted from white blood cells using TRIzol reagent. RNA quality was checked using an Agilent 2100 Bioanalyzer.

### Genotyping

SNaPshot assay (Life Technologies) was used on genomic DNA from blood of familial PTC patients for genotyping *HABP2* G534E variant (SNP rs7080536) as previously described [9] (He at al. 8q24 2009). The primers for PCR were as follows: forward 5’-TATGCCTCTGTTTCCCTTAG-3’ and reverse 5’- TGAGGTCCAGAAGACAGTAC-3’.

PCR assays were performed according to a standard protocol using AmpliTaq Gold DNA polymerase (Life Technologies) as follows: 10 min at 94°C; followed by 32 cycles of 15 s at 94°C, 15 s at 58°C, and 1 min at 72°C; followed by a final extension of 5 min at 72°C.

Screening for the SNP 7080536 in Ohio PTC patients and controls was carried out by deCODE Genetics in Reykjavik, Iceland, applying the Centaurus (Nanogen) single-track assay [13,10]. To confirm the A allele at rs7080536 as identified from single-track assay, we used SNaPshot assay (as described above) on unamplified DNA from roughly 200 samples extracted from blood and the results were 100% concordant.

### Quantitative PCR (qPCR) measurement of gene expression

One μg of RNA was reverse transcribed using the High Capacity Reverse Transcription kit (Applied Biosystems). The expression of *HABP2* was measured using PrimeTime® qPCR Assays (Hs.PT.58.592181, Hs.PT.58.39116864,and Hs.PT.58.38507028; Integrated DNA Technologies) and the expression of *GAPDH* as internal control was measured using Human GAPD (GAPDH) (Life Technologies). The real time PCR reaction was run in the ABI Prism 7900HT Sequence Detection System (Applied Biosystems). Fast TaqMan assay reaction mix was used and the conditions were as follows: 95°C for 5 min followed by 40 cycles at 95°C, 5 s and 60°C, 5 s. At the end of the assay an aliquot of the reaction was loaded on 1.5% agarose gel.

## Results

### Frequency of the G534E variant of *HABP2* in patients and controls

**Fig 1:**
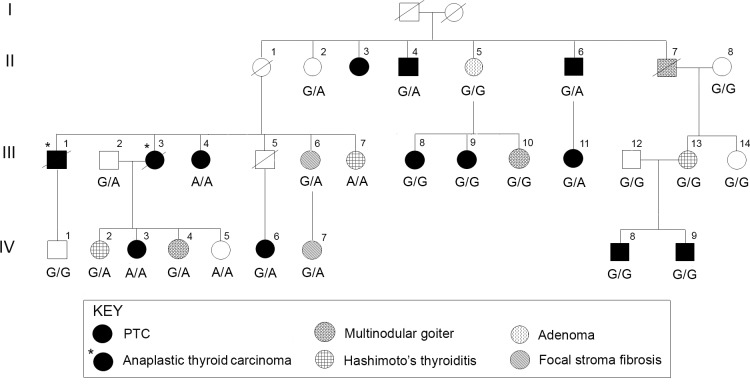
Genotyping of *HABP2* G534E variant in Family 1. The pedigree shows genotypes of the *HABP2* variant (c.1601G>A; p.G534E). Circle, female; square, male. Please note that in this family the basis of the predisposition to PTC has been determined. It is a single nucleotide variant in a long-range enhancer on chromosome 4q (He et al., 2013) [7].

Using SNaPshot analysis on one affected individual from each of the 179 available families we found 11 heterozygous variant carriers (6.1%; Table 1) corresponding to a minor allele frequency of 3.1% (Table 1). Six of the eleven variant carriers are found among the 101 families with 3 or more affected individuals (Group 1) while five are found in the remaining 75 families where only 2 individuals are affected (Group 2). We genotyped the available family members of the 8 families that had blood DNA available for at least two individuals; six belonging to Group 1 and two belonging to Group 2. One of these families (Family 1) is a large family in which after genotyping the 25 available samples the variant clearly disclosed no cosegregation with the disease (simplified pedigree shown in Fig 1). Moreover, as many as four individuals were homozygous for the variant (two with PTC–III,4 and IV,3; one with Hashimoto’s thyroiditis–III,7; and one unaffected–IV,5). There are several PTC patients in Family 1 that are not carriers of the risk allele (A) (III,8 and 9 and IV,8 and 9). Interestingly, a causative genomic alteration in this family has been described previously [7]. The mutation is a single nucleotide change located in a long-range enhancer element on chromosome 4q.

**Table 1:** Genotyping results of variant *HABP2* G534E (rs7080536) in Ohio cohorts.

		Genotype							
		GG		GA		AA		Minor Allele Frequency (%)	p-value*
Sample	Individuals	Count	Proportion (%)	Count	Proportion (%)	Count	Proportion (%)		
Familial PTC	179	168	93.9	11	6.1	0	0.0	3.1	0.32
Group 1 (3+)	103	97	94.2	6	5.8	0	0.0	2.9	0.46
Group 2 (2)	76	71	93.4	5	6.6	0	0.0	3.3	0.67
Sporadic PTC	1160	1067	92.0	91	7.8	2	0.17	4.1	0.24
Controls	1395	1274	91.3	121	8.7	0	0.0	4.3	

* Fisher's Exact test p-value, comparing genotype distributions with Controls

The genotyping results for families 1 to 6 (Fig 1 and S1 Fig) clearly show no segregation between PTC and the G534E variant. There is at least one affected individual in each family that does not carry the *HABP2* variant (marked with asterisk). The results in Family 7 and Family 8 are compatible with cosegregation but this can obviously be just a matter of chance.

Genotyping was further performed on blood DNA from 1160 sporadic PTC cases and 1395 controls (Table 1). The G534E variant is present in a total of 93 patients (8.0%), with 2 patients being homozygous for the variant (0.17%) leaving 91 heterozygous patients (7.8%). Among the controls there are 121 individuals (8.7%) heterozygous for the variant but no homozygous variant carriers. We calculated Fisher’s exact test p-value comparing genotype distributions of different groups of cases with controls and we found no statistically significant difference in distribution (Table 1). The minor allele (A) frequency is 4.1% in cases and 4.3% in controls showing no statistically significant difference between the two groups (Fisher’s exact test p-value = 0.676).

### RNA expression of *HABP2*

**Fig 2:**
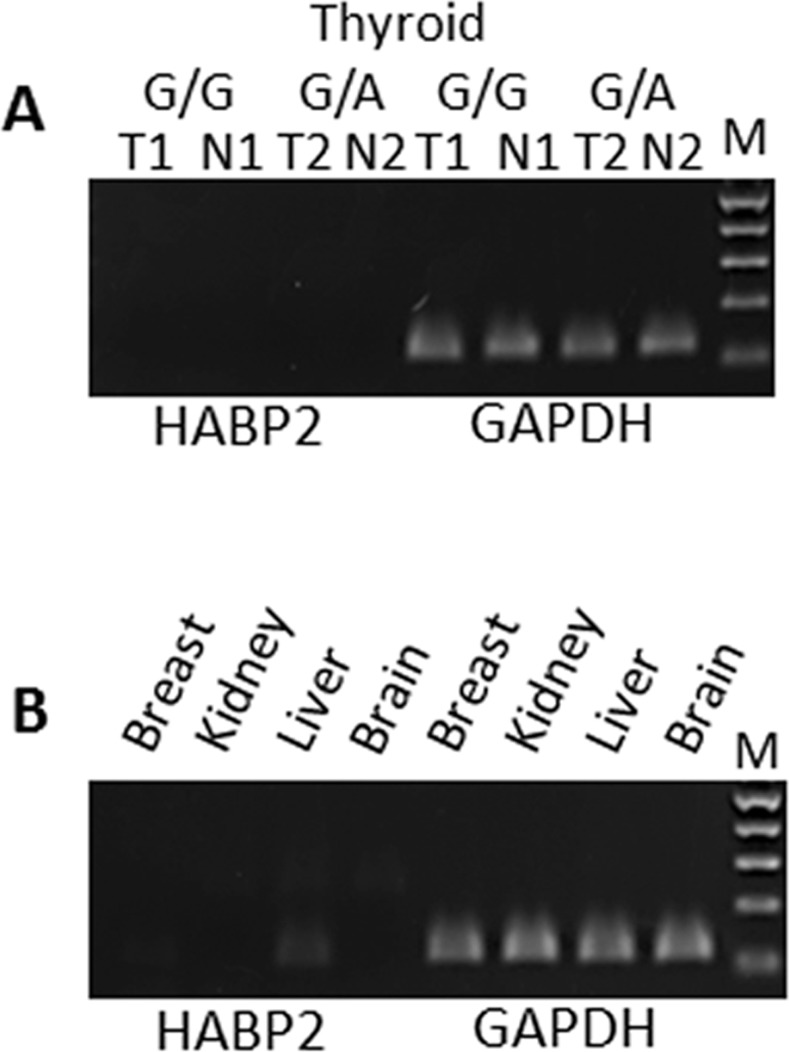
The *HABP2* gene is mainly expressed in liver and not expressed in thyroid tumors or adjacent normal thyroid tissue from patients carrying or not carrying the *HABP2* G534E variant. (A) Detection of the *HABP2* gene expression in thyroid tumor (T) or adjacent normal (N) sample. Patient 1 was heterozygous for the variant (G/A), patient 2 was homozygous wild-type (G/G). (B) qPCR in normal tissue. *HABP2* showing faint but clear expression in liver. GAPDH was used as internal control. Details for qPCR reactions are described in Materials and Methods.

Gara et al. [12] reported a higher mRNA expression of *HABP2* in the tumor tissue as compared with adjacent normal tissue. This finding is different from the data in Human Gene Atlas [14] and Illumina Body Map (Array Express accession: E-MTAB-513; http://www.ebi.ac.uk/arrayexpress/experiments/E-MTAB-513/) according to which *HABP2* mRNA is expressed primarily in liver and not expressed in normal thyroid. We studied RNA expression of the gene in fresh frozen paired tumor and normal thyroid tissue from heterozygotes for the variant (n = 3) as well as from wild-type homozygotes (n = 5), all representing sporadic PTC. *HABP2* expression was not detected in any of the samples (the Ct value was undetermined for *HABP2* while in the range of 20–22 for GAPDH endogenous control; S2 Fig). We also performed qPCR on cDNAs derived from human breast, kidney, liver, and brain tissue and expression was seen in liver with traces of expression in breast (S3 Fig). When using two different qPCR assays expression of the gene is still the highest in liver but expression also is detectable in brain and kidney (S4 Fig). Reactions were loaded on agarose gel to visualize qPCR products and results can be seen in Fig 2. On the gel the *HABP2* amplicon can be seen in liver but not in thyroid (tumor or adjacent normal tissue) that either carry or don’t carry the variant.

## Discussion

In our study we find no evidence supporting a role for the *HABP2* G534E variant (SNP rs7080536) in PTC. There is no difference in the frequency of variant carriers among familial PTC cases (grouped by the number of affected family members), sporadic PTC cases, and controls (Table 1). We found no cosegregation of the variant in six out of eight families tested. It is noticeable that in two medium size families (Family 7 and Family 8, S1 Fig) the variant is segregating with PTC and this finding is compatible with cosegregation as well as with the result of chance alone. The fact that the cosegregation occurs in only two out of eight tested families speaks in favor of our interpretation of the data, that is, that the variant is not associated with PTC.

When genotyping our cases (n = 1160) and controls (n = 1395) (mostly Caucasian; for clinical and demographic information see S1 Table) we found the minor allele frequency (MAF) to be 4.1% and 4.3% respectively. When examining the MAF of the *HABP2* G534E variant in a database of controls (http://evs.gs.washington.edu/EVS/; Table 2) we found it to be 4.0% in a European American population (n = 4300) while it was remarkably different at 0.66% in an African American population (n = 2203). In another database of controls, the 1000 Genomes Project Phase 3 (www.1000genomes.org; Table 3), MAF for the total population is 0.81% but we found pronounced differences in MAF among 5 populations with the highest MAF of 2.7% reported for the individuals of European ancestry, while the frequency is 0% in East Asian Ancestry.

**Table 2:** Genotyping results for SNP rs7080536 from NHLBI GO Exome Sequencing Project (ESP; http://evs.gs.washington.edu/EVS/).

		Genotype						
		GG		GA		AA		Minor Allele Frequency (%)
Sample	Individuals	Count	Proportion (%)	Count	Proportion (%)	Count	Proportion (%)	
European American	4300	3971	92.3	324	7.6	5	0.1	4.0
African American	2203	2174	98.7	29	1.3	0	0.0	0.66

**Table 3:** Race distribution in 1000 Genome final release Phase 3 (total of 2504 individuals) and genotyping results for SNP rs7080536. **(**http://www.1000genomes.org).

Race	Individuals		Genotype (GA or AA)		Minor Allele Frequency (%)
	Count	Proportion (%)	Count	Proportion (%)	
East Asian Ancestry (EAS)	504	20.1	0	0.0	0
South Asian Ancestry (SAS)	489	19.5	4	0.8	0.4
African Ancestry (AFR)	661	26.4	0	0.0	0
European Ancestry (EUR)**	503	20.1	26*	5.2	2.7
American Ancestry (AMR)**	347	13.9	10	2.9	1.4

*25 samples are GA, 1 sample is AA

** EUR and AMR can be grouped as White

As reported by Gara et al. [12] the SNP rs7080536 was found in 4.7% of 423 thyroid cancer patients that had the sequence of the variant nucleotide position reported in the TCGA. Thyroid cancer patients in TCGA were selected not to be familial. When analyzing the race distribution of the 507 available thyroid cancer patients we note that the majority of individuals (65.7%) can be categorized as white (Table 4). The 404 individuals with UCSC mutation calling info (TCGA database—Nov 24^th^ 2015) we find 21 heterozygous carriers of the variant, 19 of them being of European Ancestry and for two of them race is not specified. This confirms that most commonly carriers of the A allele are of European origin for PTC cases and controls (MAF in controls is 2.7% (Table 3), 4.0%(Table 2) or 4.3% (controls in Table 1)).

**Table 4:** Race distribution of 507 Thyroid Carcinoma Samples (THCA) from The Cancer Genome Atlas (09/21/15) (http://cancergenome.nih.gov/).

	Individuals	
Race	Count	Proportion (%)
Not Specified	94	18.5
American Indian or Alaska Native	1	0.2
Asian	52	10.3
Black or African American	27	5.3
White	333	65.7

Based on the results of our qPCR assays and gene expression data published in different databases, we conclude that the *HABP2* gene is not expressed in healthy thyroid or tumor thyroid tissue from sporadic PTC cases. Our genetic findings and gene expression findings allow us to say that the *HABP2* G534E variant is not a player in the predisposition to PTC.

## Supporting Information

S1 Fig Pedigrees of 7 families genotyped for the HABP2 G534E (c.1601G>A) variant in available samples. (PDF)

S2 Fig qPCR reaction in paired tumor and unaffected tissue from 8 PTC patients. (PDF)

S3 Fig qPCR reaction in normal kidney, liver, breast and brain. (PDF)

S4 Fig qPCR reaction using 2 different qPCR assays in normal kidney, liver, breast and brain. (PDF)

S1 Table Clinical and demographic information on cases and controls. (PDF)
