# Supplementary material for: HABP2 G534E Variant in Papillary Thyroid Carcinoma
Source: PLoS One. 2016 Jan 8;11(1):e0146315. doi: 10.1371/journal.pone.0146315 (PMC4706330; doi:10.1371/journal.pone.0146315)
Supplement: S1 Fig — (PDF) [file pone.0146315.s001.pdf]

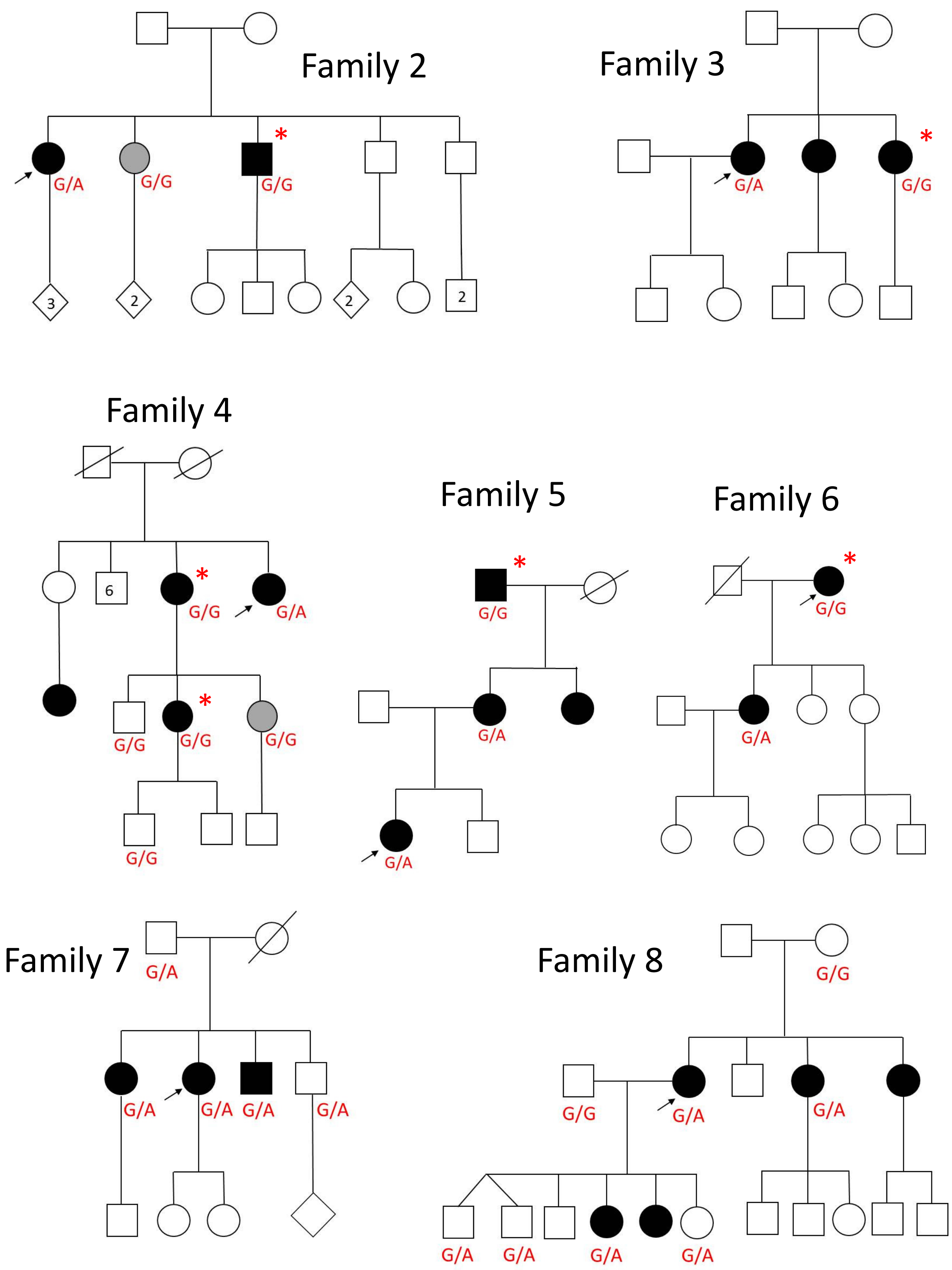

S1 Fig. Pedigrees of 7 families genotyped for the HABP2 G534E (c.1601G>A) variant in available samples. Squares, male family member; circles, female family member; diamond, gender not available; arrow, proband; black symbols, PTC; grey symbols, goiter; empty symbols, unaffected; asterisk, PTC not carrying the variant.
