# Supplementary material for: HABP2 G534E Variant in Papillary Thyroid Carcinoma
Source: PLoS One. 2016 Jan 8;11(1):e0146315. doi: 10.1371/journal.pone.0146315 (PMC4706330; doi:10.1371/journal.pone.0146315)
Supplement: S2 Fig — (PDF) [file pone.0146315.s002.pdf]

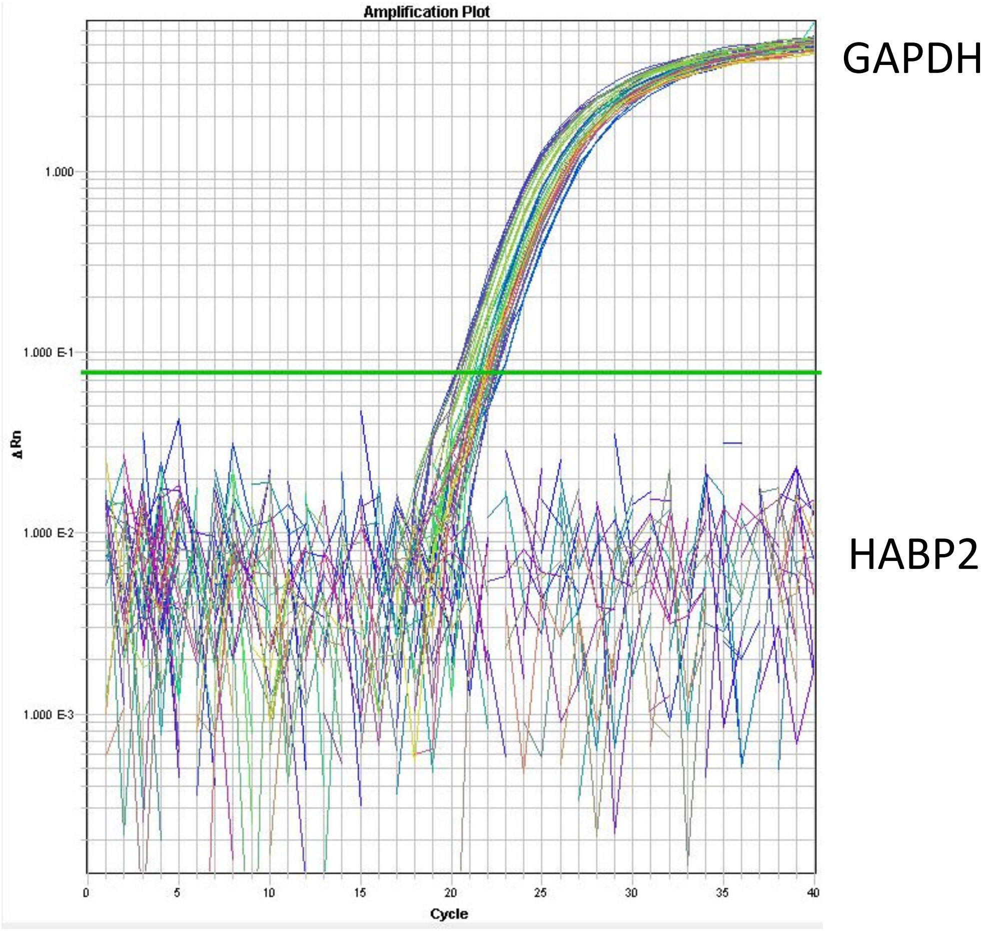

S2 Fig. qPCR reaction in paired tumor and unaffected tissue from 8 PTC patients. cDNA was synthesized on total RNA extracted from samples. All samples show the expression of internal control (GAPDH) while no expression can be detected for HABP2. The same result was obtained with all 3 PrimeTime® qPCR assays (listed in Materials and Methods).
