# Supplementary material for: HABP2 G534E Variant in Papillary Thyroid Carcinoma
Source: PLoS One. 2016 Jan 8;11(1):e0146315. doi: 10.1371/journal.pone.0146315 (PMC4706330; doi:10.1371/journal.pone.0146315)
Supplement: S3 Fig — (PDF) [file pone.0146315.s003.pdf]

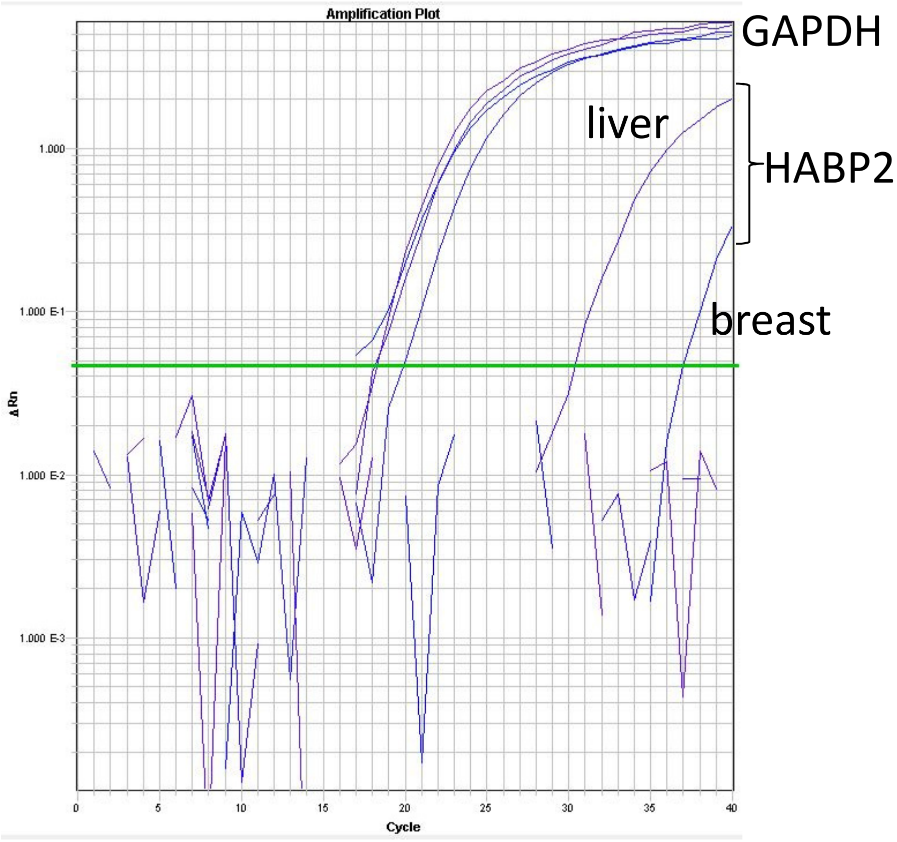

S3 Fig. qPCR reaction in normal kidney, liver, breast and brain. cDNA was synthesized on total RNA extracted from samples. PrimeTime® qPCR assay Hs.PT.58.592181 was used to test for HABP2 expression. GAPDH used as internal control was expressed in all samples.
