# Supplementary material for: HABP2 G534E Variant in Papillary Thyroid Carcinoma
Source: PLoS One. 2016 Jan 8;11(1):e0146315. doi: 10.1371/journal.pone.0146315 (PMC4706330; doi:10.1371/journal.pone.0146315)
Supplement: S4 Fig — (PDF) [file pone.0146315.s004.pdf]

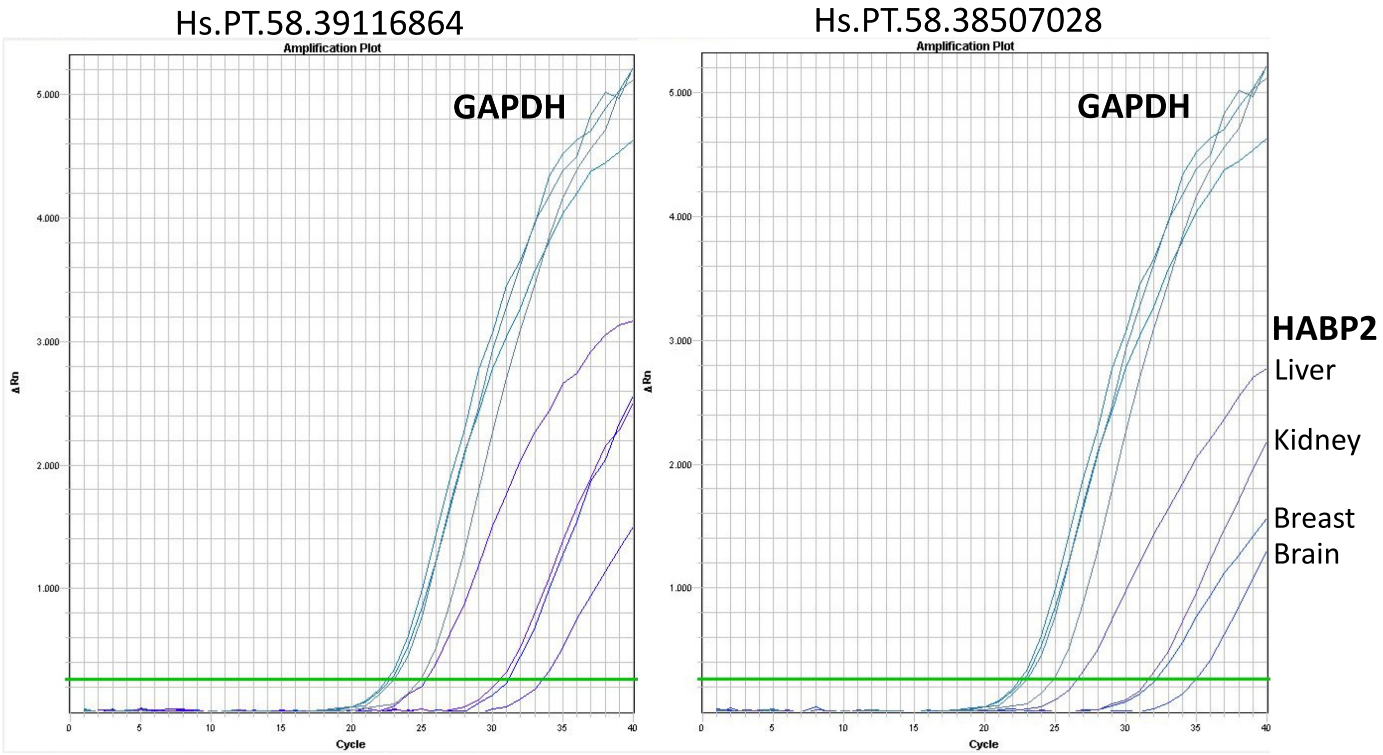

S4 Fig. qPCR reaction using 2 different qPCR assays in normal kidney, liver, breast and brain. GAPDH used as internal control was expressed in all samples. Small differences in HABP2 expression in different organs can be seen between these two assays and the first assay used. HABP2 still has the highest expression in liver.
