# Supplementary material for: HABP2 G534E Variant in Papillary Thyroid Carcinoma
Source: PLoS One. 2016 Jan 8;11(1):e0146315. doi: 10.1371/journal.pone.0146315 (PMC4706330; doi:10.1371/journal.pone.0146315)
Supplement: S1 Table — (PDF) [file pone.0146315.s005.pdf]

S1 Table. Clinical and demographic information on cases and controls

	Cases N (%)	Controls N (%)
**Gender**		
**Female**	898 (77)	1069 (76)
**Male**	272 (23)	335 (24)
**Race**		
**Caucasian**	1097 (93.8)	1317 (93.8)
**African American**	42 (3.6)	54 (3.8)
**Asian**	31 (2.6)	33 (2.4)
**Mean age^**	41.4 yrs (range 7-88 yrs)	43.8 yrs (range 18-87 yrs)
**Histologic sub-type**		
**PTC, classic type**	680	
**PTC, follicular variant**	240	
**microPTC**	207	
**PTC, other**	43	
**Total**	1170 (100)	1404(100)

^ Mean age of cases is age at diagnosis of thyroid cancer; mean age for controls is age at time of study enrollment and blood draw.

PTC = papillary thyroid carcinoma; microPTC = PTC less than 1.0 cm in dimension
